# Increased Thioredoxin-1 Expression Promotes Cancer Progression and Predicts Poor Prognosis in Patients with Gastric Cancer

**DOI:** 10.1155/2019/9291683

**Published:** 2019-02-18

**Authors:** Wenjing Shang, Zhongdong Xie, Fengying Lu, Daoquan Fang, Tianbin Tang, Ruichun Bi, Lingli Chen, Lei Jiang

**Affiliations:** ^1^Central Laboratory, The First Affiliated Hospital of Wenzhou Medical University, Wenzhou 325000, China; ^2^Department of Gastrointestinal Surgery, The First Affiliated Hospital of Wenzhou Medical University, Wenzhou 325000, China; ^3^Department of Neurology, The First Affiliated Hospital of Wenzhou Medical University, Wenzhou 325000, China

## Abstract

**Background:**

Thioredoxin-1 (Trx-1) is a small redox protein, which plays an important role in many biological processes. Although increased expression of Trx-1 in various solid tumors has been reported, the prognostic significance and function of Trx-1 in human gastric cancer (GC) are still unclear. Here, we investigated the clinical and prognostic significance of Trx-1 expression and the function and mechanism of Trx-1 in human GC.

**Methods:**

We analyzed Trx-1 mRNA expression from the GEO database and Trx-1 protein expression in 144 GC tissues using immunohistochemistry. Effects of Trx-1 on GC cell were assessed *in vitro* and *in vivo* through Trx-1 knockdown or overexpression. The antitumor effects of the Trx-1 inhibitor, PX-12, on GC cells were investigated. PTEN and p-AKT expressions were evaluated by Western blotting.

**Results:**

Increased Trx-1 expression was found in GC tissues and associated with poor prognosis and aggressive clinicopathological characteristics in patients with GC. High Trx-1 expression predicted poor prognosis, and its expression was an independent prognostic factor for overall survival of GC patients. Knockdown of Trx-1 expression inhibited GC cell growth, migration, and invasion *in vitro* and tumor growth and lung metastasis *in vivo*. Conversely, overexpression of Trx-1 promoted GC cell growth, migration, and invasion. We also found that PX-12 inhibited GC cell growth, migration, and invasion. Overexpression of Trx-1 caused a decrease in PTEN and increase in p-AKT levels whereas silencing Trx-1 caused an increase in PTEN and decrease in p-AKT levels in GC cells. Inhibition of AKT signaling pathway by MK2206 also inhibited GC cell growth, migration, and invasion.

**Conclusion:**

Our results indicate that Trx-1 may be a promising prognostic indicator and therapeutic target for GC patients.

## 1. Introduction

Gastric cancer (GC) is the fourth most diagnosed cancer and the second leading cause of cancer-related death worldwide [1]. 70% of GC deaths occur in developing countries with China accounting for approximately 40% of them [2]. GC is a multifactorial disease with complex reasons including *H. pylori* infection, genetics, poor lifestyle, and environmental factors [3]. Although with significant advances in surgical techniques, diagnosis, and new chemotherapy approaches, the prognosis of patients with advanced GC is poor, with a five-year survival of 5–20% and a median overall survival of 10 months [4]. Therefore, there is an urgent need to explore new diagnostic and prognostic biomarkers and effective therapeutic targets for GC patients.

Thioredoxin-1 (Trx-1) is a member of the thioredoxin protein family, which are low molecular weight (10–12 kDa) redox proteins found in both prokaryotic and eukaryotic cells [5]. Trx-1 is often upregulated in many human cancers involving the lung [6, 7], breast [8], liver [9, 10], colon and rectum [11, 12], uterine cervix [13], pancreas [14, 15], and stomach [16, 17]. Its overexpression is associated with cancer cell proliferation, inhibition of apoptosis, tumor aggressiveness, and poor prognosis in patients [18, 19]. Trx-1 interacts with a number of transcription factors, for example, nuclear factor kappa B (NF-*κ*B), activator protein-1 (AP-1), p53, and SP-1, all of which seem to regulate cell growth and survival [20–22]. Trx-1 also binds to redox-sensitive enzymes to regulate their activity including apoptosis signal-regulating kinase-1 (ASK-1) [23] and protein kinase C (*δ*, *ω*, and *ζ*) [24]. We also recently demonstrated a novel positive feedback loop between Trx-1 and S100P, which promotes colorectal cancer cell epithelial-mesenchymal transition, invasion, and metastasis by upregulating S100A4 through AKT activation [12, 25]. However, the prognostic significance and function of Trx-1 in human GC are still unclear.

In this study, we evaluated the clinical and prognostic significance of Trx-1 expression in human GC and investigated the function and molecular mechanism of Trx-1 in GC. We also examine the antitumor effects of Trx-1 inhibitor PX-12 on GC cells in vitro and demonstrated that Trx-1 promoted GC progression via activation of AKT signaling pathway. Taken together, we report that Trx-1 plays a vital role in the progression of GC and could be a significant potential therapeutic target for the treatment of GC.

## 2. Materials and Methods

### 2.1. Genomic Data Mining

Raw data from two data sets (GSE13911 and GSE15460) were downloaded from the Gene Expression Omnibus (GEO) database (https://www.ncbi.nlm.nih.gov/geo/). Each data set was examined using the Affymetrix plus 2.0 platform (Santa Clara, CA, USA), and the corresponding gene expression profiles were extracted with the fRMA package in the R 3.2.0 environment.

### 2.2. Patient Recruitment and Immunohistochemistry (IHC)

In this study, 144 stomach adenocarcinoma cases of patients were retrospectively selected from the surgical pathological database of the First Affiliated Hospital of Wenzhou Medical University, China, between 2004 and 2008. All patients received curative surgery at the First Affiliated Hospital of Wenzhou Medical University and their clinical characteristics are shown in Table 1. According to WHO histological classification, there were 93 cases of tubular adenocarcinoma, two cases of papillary adenocarcinoma, five cases of mucinous adenocarcinoma, 43 cases of poorly cohesive, and one mixed adenocarcinomas. According to the Lauren classification, 100 cases were intestinal type and 44 cases were diffuse type. One case was well differentiated, 58 moderately differentiated, and 81 poorly differentiated by pathological grading. The paraffin-embedded blocks from 144 GC patients were then cut into 4 mm sections for immunostaining and used for the IHC analysis. Written informed consent was obtained from each patient. None of the patients received radio- or chemotherapy prior to surgery. Patients who died of causes other than GC were excluded from the study. This study was approved by the Ethics Committee of the First Affiliated Hospital of Wenzhou Medical University.

Anti-human Trx-1 (Cat. # ab26320; Abcam, Cambridge, UK) was used at a concentration of 1 : 1000 for IHC. The immunostaining protocol was based on the manufacturer's recommendations. Antigen retrieval was performed using citrate buffer (pH 6.0). Trx-1 protein expression was semiquantitated using the *H*-score method as previously reported [12, 26]. The intensity of protein staining of Trx-1 (0, 1+, 2+, and 3+) and the total percentage of positively stained epithelial cells were independently scored by two investigators who were blinded to the patient's condition. A minimum of 100 cells was evaluated to calculate the IHC score. The equation: IHC score = 1 × (% 1+) + 2 × (% 2+) + 3 × (% 3+), was used to calculate the IHC score for each specimen. The IHC scores from the two investigators were then averaged and analyzed. If there is discrepancy between the two scores, a conclusive agreement was reached with the involvement of a third investigator. The association between Trx-1 expression and survival outcome was analyzed by an investigator who did not participate in the scoring process.

### 2.3. Cell Culture and Chemicals

Human GC cell lines including AGS, BGC-823, SGC-7901, KATO III, and normal gastric mucosa cell line GES-1 were purchased from the Typical Culture Collection of the Chinese Academy of Sciences, Shanghai, China. They were maintained in RPMI 1640 supplemented with 10% fetal bovine serum (FBS) (Thermo Fisher Scientific, Waltham, MA, USA) in a 37°C incubator supplemented with 5% CO_2_. PX-12 (1-methylpropyl 2-imidazolyl disulfide, Cat. # M5342) was purchased from Sigma-Aldrich (Billerica, MA, USA). MK2206 (Cat. # S1177) was purchased from Selleck Chemicals (Houston, TX, USA).

### 2.4. Lentiviral Vectors and Transduction

Lentiviral vectors expressing enhanced green fluorescent protein (GFP) or Trx-1 gene and expressing shRNA targeting Trx-1 (shTrx-1) or firefly luciferase (shLuc) were constructed as previously described [12]. Lentiviral particles were produced in HEK293T cells by transfection of lentiviral expression vectors, pMD2.G, pMDL-G/P-RRE, and pRSV-REV as described [27]. A total of 2.5 × 10^5^ GC cells were seeded in 6-well plates and transduced with the lentivirus in the presence of 8 *μ*g/mL polybrene (Sigma-Aldrich; Billerica, MA, USA).

### 2.5. Quantitative Polymerase Chain Reaction (PCR)

Total RNA was isolated from cells using TRIzol (Invitrogen, Carlsbad, CA), and cDNA synthesis was performed using the RevertAid First Strand cDNA Synthesis Kit (Thermo Scientific) according to the manufacturer's protocol. Quantitative PCR analysis was prepared using the SYBR Premix Ex Taq (Takara, Japan). Real-time PCR was carried out utilizing the 7500 real-time PCR system (Applied Biosystems, Warrington, UK). After an initial activation step of 95°C for 10 min, 40 PCR cycles were performed using the following conditions: denaturation at 95°C for 15 s and annealing/extension at 60°C for 1 min. The relative quantitation was expressed in Ct values, which were determined for triplicate reactions for each target gene and GAPDH. Triplicate Ct values were averaged and the GAPDH Ct subtracted to obtain ΔCt. The fold change of the treated target gene relative to the control was calculated as 2^−ΔΔCt^. The following forward and reverse primers were used, respectively: Trx-1: 5′-CAA CCC TTT CTT TCA TTC CCT CT -3′ and 5′-CAC CCA CCT TTT GTC CCT TCT-3′ and GAPDH: 5′-CCA GCC GAG CCA CAT CGC TC-3′ and 5′-ATG AGC CCC AGC CTT CTC CAT-3′.

### 2.6. Western Blot Analysis

Cells were lysed in RIPA buffer (Thermo Fisher Scientific) containing protease and phosphatase inhibitor (Thermo Fisher Scientific). The concentration of total protein was measured by the Pierce BCA Protein Assay Kit (Thermo Fisher Scientific). Total protein (50 *μ*g) was separated by SDS-PAGE using a 12% gel and subsequently transferred onto polyvinylidene difluoride membranes (EMD Millipore, Billerica, MA, USA). Membranes were blocked in 5% albumin from bovine serum (BSA, Biosharp, China) for 2 h at room temperature and incubated with primary antibodies overnight at 4°C. Subsequently, the membranes were incubated with the appropriate horseradish peroxidase-conjugated secondary antibody for 1 h at room temperature. Antibody binding signals were detected using an enhanced chemiluminescence detection system (Bio-Rad, California, CA, USA). The following primary antibodies were used: rabbit anti-Trx-1 (diluted 1 : 10,000; Cat. # ab26320; Abcam, Cambridge, UK), rabbit anti-GAPDH (diluted 1 : 1000; Cat. # 5174S; Cell Signaling Technology, Danvers, MA, USA), rabbit anti-*β*-actin (diluted 1 : 1000; Cat. # 4970, Cell Signaling Technology), rabbit anti-phosphatase and tensin homolog (PTEN) (diluted 1 : 5000; Cat. # ab32199; Abcam), rabbit anti-AKT (diluted 1 : 1000; Cat. # 4691; Cell Signaling Technology), and rabbit anti-phosphorylated AKT (p-AKT) (diluted 1 : 2000; Cat. # 4060; Cell Signaling Technology). The secondary antibody was goat anti-rabbit IgG (H+L) HRP conjugated (diluted 1 : 5000; Cat. # GAR007; Multi Sciences, China).

### 2.7. Cell Proliferation Assays

The cell counting kit-8 (CCK-8) assay was used to examine cell viability and proliferation. Cells (2000 cells/well) were seeded in 96-well plates with a volume of 100 *μ*L complete medium. 10 *μ*L of CCK8 (Dojindo, Japan) solution was added to each well at the indicated time points (0, 24, 48, 72, and 96 h). After incubation for 4 h, the absorbance value (OD) was measured at 450 nm.

For the colony formation assay, GC cells were counted and inoculated in 6-well plates (500 cells/well). The culture medium RPMI 1640 containing 10% FBS was replaced every 3 days. After 14 days, cells were fixed for 20 min with 4% paraformaldehyde and stained with 0.1% crystal violet solution for 5 min. The numbers of colonies were counted. The experiment was repeated three times.

### 2.8. Cytotoxicity Assays

For toxicity assays, GC cells (5000 cells/mL) were seeded in 96-well plates (100 *μ*L/well). After cell attachment, cells were treated with PX-12 (Trx-1 inhibitor) or MK2206 (AKT inhibitor) in complete medium for 24 or 48 hours. Cell viability was determined by the CCK-8 assay.

### 2.9. Apoptosis Assay

Cell apoptosis was measured by an annexin V-FITC apoptosis detection kit (Multi Sciences, Hangzhou, China) according to the manufacturer's protocols. Briefly, cells cultured in 6-well plates were trypsinized and collected by centrifugation. Each cell pellet was washed twice with cold PBS and resuspended in binding buffer at a density of 1 × 10^6^ cells/mL. Then cells were stained with annexin V-FITC (5 *μ*L) and PI (1 *μ*g/mL), and cell apoptosis was analyzed with the FACSCalibur flow cytometer (Becton Dickinson, Franklin Lakes, NJ, USA).

### 2.10. Cell Migration and Invasion Assays

Cell migration and invasion assays were performed in 24-well Transwell cell culture chambers with 8 *μ*m pores (Costar; Corning Incorporated, Cambridge, MA, USA) according to the manufacturer's instructions. For the migration assay, AGS and BGC-823 cells (1.5 × 10^5^/200 *μ*L) were seeded onto the Transwell filter membrane chambers in a medium without FBS. A medium supplemented with 20% FBS was added to the lower chambers as a chemoattractant. After being incubated at 37°C for 16 h (for AGS cells) or 22 h (for BGC-823 cells), cells in the lower chambers were fixed with 4% paraformaldehyde and stained with 0.1% crystal violet solution. Cells that did not migrate were removed from the upper chamber surface using a cotton swab, and the number of cells that migrated to the lower chamber was counted in 5 fields (fields were randomly selected under a light microscope at magnification, ×20). For the invasion assay, Transwell membranes were precoated with 10 *μ*L of Matrigel (4.53 mg/mL; BD Biosciences, San Jose, CA, USA) prior to the process described above. AGS and BGC-823 cells were incubated for 18 h and 22 h, respectively.

### 2.11. Tumor Growth and Lung Metastasis in Nude Mice

All animal experiments were approved by the Animal Experimental Ethics Committee of Wenzhou Medical University. Four- to six-week-old female athymic nude mice were purchased from the Shanghai SLAC Laboratory Animal Co. Ltd., Shanghai, China. The mice were given one week to adapt to the new environment before further experimentation. Sixteen nude mice were randomly divided into two groups and were injected subcutaneously with 5 × 10^5^ BGC-823 cells transduced with lenti-shLuc or lenti-shTrx-1. Tumor length and width were measured using a vernier caliper every 3 days. The volume of the tumor (mm^3^) was calculated using the following formula: 0.5 × length × width^2^. We also evaluated the metastatic capability of GC cells by lung metastasis in athymic nude mice (*n* = 8 per group). Briefly, 1 × 10^6^ BGC-823 cells transduced with lenti-shLuc or lenti-shTrx-1 suspended in 200 *μ*L PBS were injected into the tail vein of athymic nude mice (*n* = 8 per group). The body weight of mice was measured every 3 days. Five weeks later, the mice were sacrificed by cervical vertebra dislocation and lung metastases were evaluated.

### 2.12. Statistical Analysis

Data are presented as mean ± SD. Enumerated data were compared using the chi-square test, and comparisons of the continuous data between the two groups were tested using an independent *t*-test (GraphPad Prism, San Diego, CA, USA). Categorical data such as sex and the tumor differentiation grades were compared and analyzed using the chi-square test or Mann-Whitney *U* test. For survival analysis, patient subgroups divided with high or low Trx-1 expression according to the median Trx-1 protein expression level were compared using the Kaplan-Meier method and univariate and multivariate Cox proportional hazards models. The log-rank test was used to assess the statistical significance of the Kaplan-Meier curves. All statistical tests were two-sided. *P* value of less than 0.05 was considered to be statistically significant.

## 3. Results

### 3.1. Clinicopathological Significance of Trx-1 Expression in GC Patients

**Figure 1:**
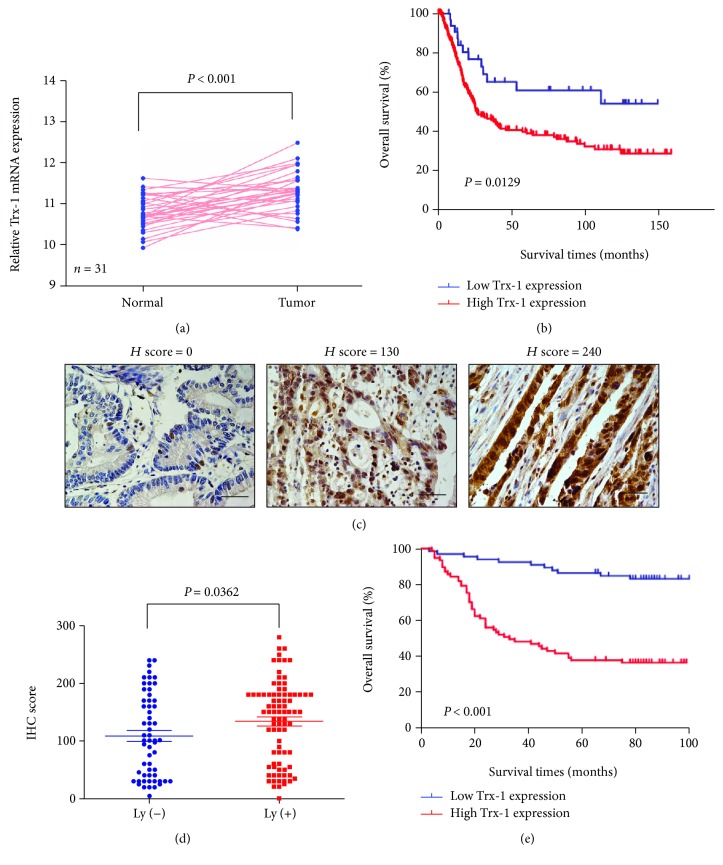
Trx-1 upregulation is associated with poor prognosis in GC. (a) Using the GSE13911 data set, differences in the Trx-1 mRNA expression levels were explored between the GC and normal tissues. (b) In the GSE15460 cohort, the patients were dichotomized into subgroups with high or low Trx-1 mRNA expression according to the Trx-1 expression level cutoff value across the cohort. The Kaplan-Meier curves were compared using the log-rank test. The median survival time was 34 months for the high Trx-1 expression group and >150 months for the low Trx-1 expression group. (c) Representative immunostaining of Trx-1 in gastric cancer tissues. Bar, 100 *μ*m. (d) Significant upregulation of Trx-1 protein expression was observed in GC with lymph node metastases relative to GC without lymph node metastasis. (e) Kaplan-Meier curves for GC patients demonstrating that those with high Trx-1 expression had a shorter survival time compared with low Trx-1-expression. The median survival time was 31 months for the high Trx-1 expression group and >100 months for the low Trx-1 expression group. GC: gastric cancer. *P* value is presented in the figures.

To explore the expression pattern of Trx-1 in GC, we analyzed Trx-1 mRNA expression data from 31 GC patients using the GSE13911 GC data set. The expression of Trx-1 in GC tissue was significantly higher than that in matched normal tissue (Figure 1(a)). We also analyzed the association between Trx-1 mRNA expression and patient survival using GSE15460 obtained from the GEO database and found that patients with high Trx-1 expression levels had shorter postoperative survival time than patients with low Trx-1 expression levels (*P* = 0.0176, Figure 1(b)). Furthermore, using immunohistochemistry for analyzing Trx-1 protein expression in GC (Figure 1(c)), significant upregulation of Trx-1 protein expression was revealed in GC with lymph node metastasis compared with GC without lymph node metastasis (*P* = 0.036, Figure 1(d)). Kaplan-Meier analysis showed that lower Trx-1 protein expression was linked to markedly longer overall survival of GC patients (*P* < 0.001, Figure 1(e)). The relationships between Trx-1 expression and the clinicopathological parameters in 144 GC patients are presented in Table 1. Correlation analysis demonstrated that high Trx-1 was significantly correlated with clinical stage (*P* = 0.029), tumor stage (*P* = 0.003), and tumor size (*P* < 0.001, Table 1 **)**.

We conducted a univariable and multivariable Cox proportional hazards regression analysis to demonstrate Trx-1's impact on predicting the survival of GC patients' postsurgical resection. The univariable analysis showed that Trx-1 protein expression, clinical stage, differentiation grade, tumor size, and tumor stage were associated with prognosis. The multivariable Cox proportional hazards regression analysis showed that after controlling for the confounding effects of age, sex, clinical stage, differentiation grade, tumor stage, and tumor size, Trx-1 expression was an independent prognostic factor for the overall survival of GC patients (Table 2). Taken together, these results consistently suggest that Trx-1 is upregulated in GC tissues and predicts a worse prognosis for GC patients.

### 3.2. Overexpression of Trx-1 Promotes Gastric Cancer Cell Proliferation, Migration, and Invasion

**Figure 2:**
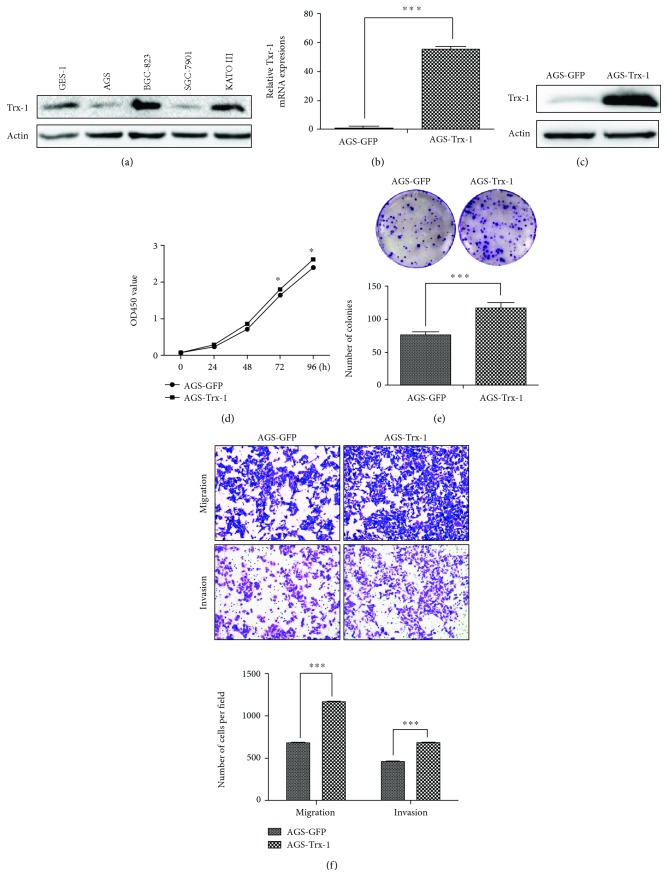
Overexpression of Trx-1 promotes gastric cancer cell growth, colony formation, invasion, and migration. (a) The expression level of Trx-1 was analyzed by Western blotting in GES-1, AGS, BGC-823, SGC-7901, and KATO III cell lines. (b) Increased mRNA level of Trx-1 in AGS cells transduced with lenti-Trx-1 using quantitative RT-PCR. (c) Increased protein level of Trx-1 in AGS cells transduced with lenti-Trx-1 revealed by Western blotting. (d) Ectopic expression of Trx-1 promoted AGS cell growth. Cell growth was assessed using the CCK-8 assay. (e) Ectopic expression of Trx-1 promoted AGS cell plate colony formation. (f) Ectopic expression of Trx-1 enhanced AGS cell migration and invasion via the Transwell assay. Representative images from triplicate experiments are shown. Magnification, ×200. The quantitation of migrated and invaded cells is shown in the bottom panel. ^∗^ *P* < 0.05, ^∗∗∗^ *P* < 0.001.

The protein expression levels of Trx-1 in 4 GC cell lines and 1 normal mucosa cell line were demonstrated by Western blotting (Figure 2(a)). Based on those results, we overexpressed the Trx-1 gene in the AGS cell line that exhibited a low level of Trx-1 while silencing Trx-1 expression in the BGC-823 cell line that exhibited a high Trx-1 expression. The increased mRNA and protein level of Trx-1 was verified in AGS cells transduced with lenti-Trx-1 (AGS-Trx-1) compared with control AGS cells transduced with lenti-GFP (AGS-GFP) using qRT-PCR (Figure 2(b)) and Western blotting (Figure 2(c)). The CCK-8 and plate colony formation assays showed that overexpression of Trx-1 promoted AGS cell growth (Figures 2(d) and 2(e)). We also evaluated the effects of Trx-1 on cell migration and invasion using a Transwell assay. As shown in Figure 2(f), overexpression of Trx-1 promoted migration and invasion of AGS cells.

### 3.3. Suppression of Trx-1 Expression Inhibits Gastric Cancer Cell Proliferation, Migration, and Invasion

**Figure 3:**
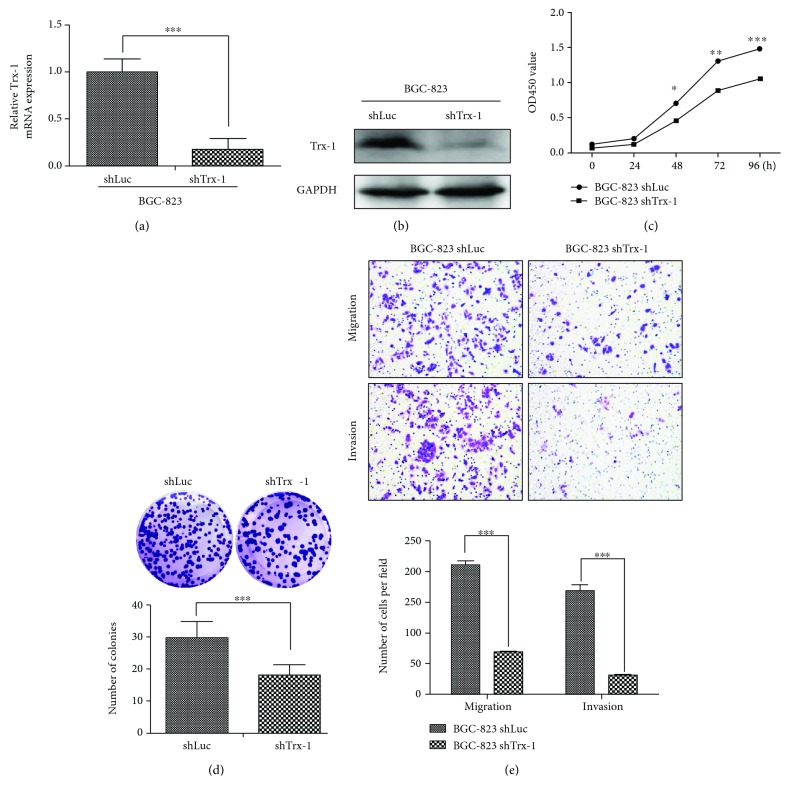
Downregulation of Trx-1 by lentiviral vector-mediated RNAi inhibits gastric cancer cell growth, clone formation, invasion and migration. (a) Decreased Trx-1 mRNA level in BGC-823 cells transduced with lenti-shTrx-1 revealed by quantitative RT-PCR. (b) Decreased Trx-1 protein level in BGC-823 cell transduced with lenti-shTrx-1 revealed by Western blotting. (c) Downregulation of Trx-1 inhibited BGC-823 cell growth as revealed by CCK-8 assay. (d) Downregulation of Trx-1 inhibited BGC-823 cell plate colony formation. (e) Downregulation of Trx-1 inhibited BGC-823 cell migration and invasion. Representative images from triplicate experiments are shown. Magnification, ×200. The quantitation of migrated and invaded cells is shown in the bottom panel. ^∗^ *P* < 0.05, ^∗∗^ *P* < 0.01, ^∗∗∗^ *P* < 0.001.

To study the biological function of Trx-1 in GC, we constructed a stable BGC-823 cell line with Trx-1 knockdown (BGC-823-shTrx-1) using a lentivirus carrying a shRNA targeting the Trx-1 gene (lenti-shTrx-1). Quantitative RT-PCR and Western blot analysis showed that lenti-shTrx-1 had a strong inhibitory effect on Trx-1 mRNA and protein expression (Figures 3(a) and 3(b)). As shown in Figures 3(c) and 3(d), knockdown of Trx-1 by shRNA significantly inhibited BGC-823 cell growth by CCK-8 and in colony formation assays. In addition, suppression of Trx-1 with lenti-shTrx-1 inhibited BGC-823 cell migration and invasion (Figure 3(e)). We also knocked down Trx-1 expression by lent-shTrx-1 in KATO III and AGS cells. As shown in Supplementary Figure S1, knockdown of Trx-1 inhibited KATO III and AGS cell proliferation, migration, and invasion.

### 3.4. Effects of Trx-1 on Gastric Cancer Growth and Metastasis *In Vivo*

**Figure 4:**
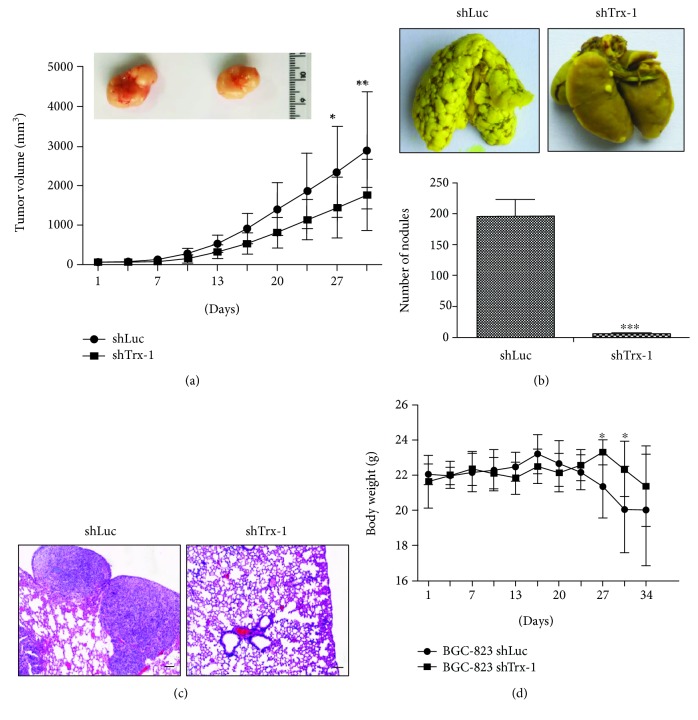
Knockdown of Trx-1 inhibits gastric cancer cell growth and metastasis *in vivo*. (a) Reduced tumor volume of xenografts generated by BGC-823 cells transduced with lenti-shTrx-1 compared with those transduced with lenti-shLuc (*n* = 8 per group). (b) Knockdown of Trx-1 inhibited lung metastases of BGC-823 cells. Representative photographs showing macroscopic appearances of lung metastasis. Metastatic tumor nodules were identified as whitish and patchy areas. The number of metastatic nodules for the two groups is shown in the bottom panel. ^∗∗∗^ *P* < 0.001. (c) H&E staining shows the lung nodules from BGC-823 cells transduced with lenti-shLuc (control) or lenti-shTrx-1. Bar, 100 *μ*m. (d) The mouse body weight was measured after tail vein injection with BGC-823-shLuc or BGC-823-shTrx-1 cells (*n* = 8 per group). ^∗^ *P* < 0.05.

To further evaluate the effects of Trx-1 on the tumorigenicity of GC cells in nude mice, BGC-823 cells transduced with lenti-shLuc or lenti-shTrx-1 were subcutaneously injected into the flanks. As shown in Figure 4(a), tumor derived from BGC-823 cells transduced with lenti-shTrx-1 grew slower than that derived from the control group (BGC-823-shLuc). For the *in vivo* metastasis experiment, five weeks after tail vein injection with BGC-823-shLuc or BGC-823-shTrx-1 cells, macroscopic nodules on lung surfaces were found in both groups of mice. However, knockdown of Trx-1 in BGC-823 cells showed a significant decrease in the total number of tumor nodules in the lungs of mice compared with control (Figure 4(b)). H&E staining confirmed that metastatic tumor nodules were formed by adenocarcinoma cells and the suppression of Trx-1 reduced the number of tumor nodules (Figure 4(c)). The body weight of the control group mice was significantly decreased compared to that of the shTrx-1 group at 27 and 30 days (Figure 4(d)). These results suggest that the knockdown of Trx-1 inhibited GC cell growth and metastasis *in vivo*.

### 3.5. Trx-1 Inhibitor, PX-12, Inhibits AGS and BGC-823 Cell Growth, Migration, and Invasion

**Figure 5:**
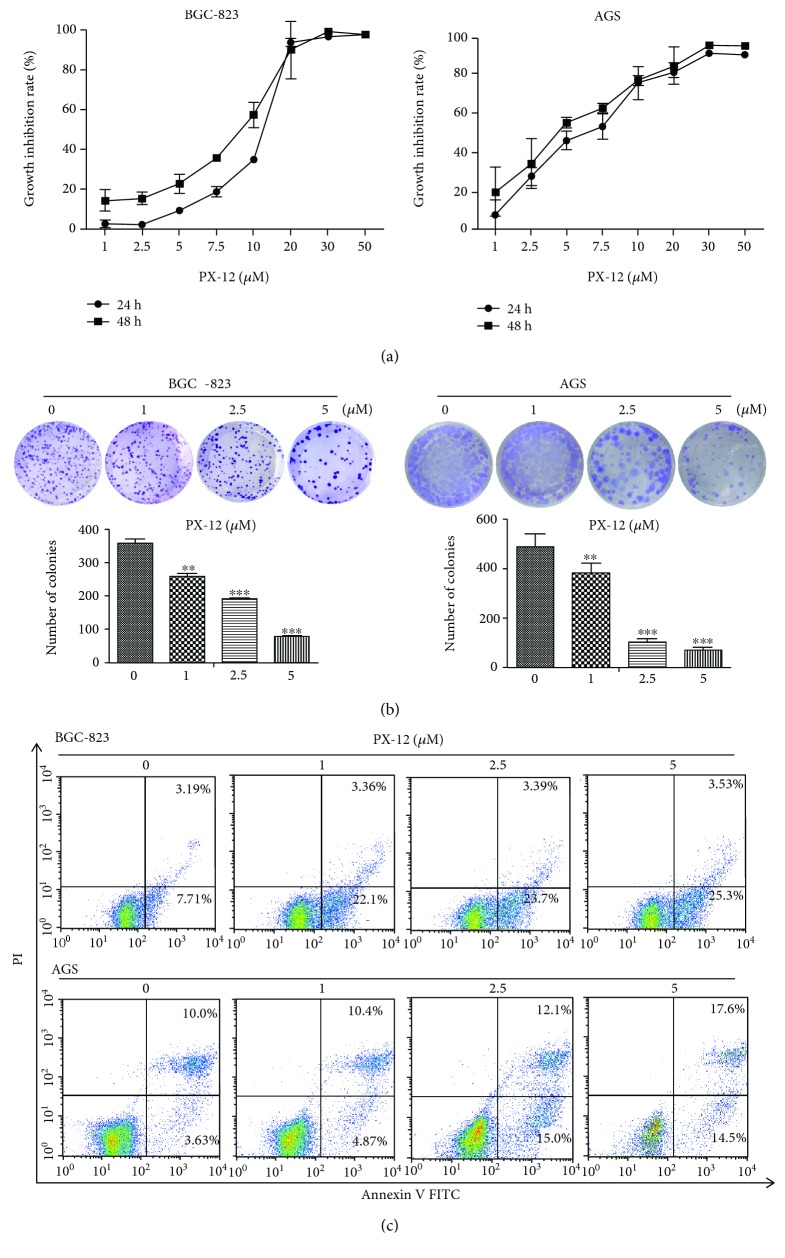
Effects of Trx-1 inhibitor, PX-12, on BGC-823 and AGS cell growth and apoptosis. (a) BGC-823 and AGS cells were treated with PX-12 in increasing concentrations for 24 and 48 hours. 24-hour IC_50_ = 11.59 *μ*М, 48-hour IC_50_ = 8.80 *μ*М for BGC-823 cells and 24 h IC_50_ = 6.43 *μ*М, 48 h IC_50_ = 4.43 *μ*М for AGS cells. (b) Gastric cancer cell colony formation is affected by PX-12 treatment. BGC-823 and AGS cells were treated with the indicated concentrations of PX-12 (0, 1, 2.5, and 5 *μ*M) for 48 hours and then changed to medium not containing PX-12 to allow for colony formation for 14 days. (c) Apoptosis analysis by annexin V/PI staining in BGC-823 and AGS cells treated with PX-12 (0, 1, 5, and 10 *μ*M) for 48 hours.

**Figure 6:**
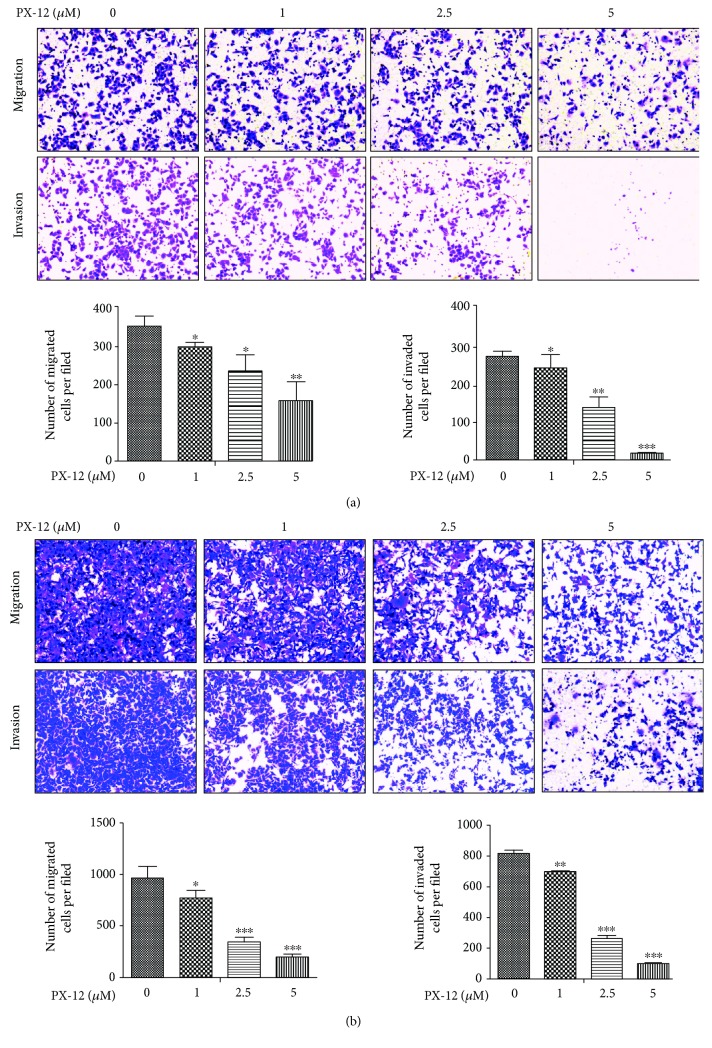
Effects of Trx-1 inhibitor, PX-12, on gastric cancer cell migration and invasion. (a) PX-12 treatment inhibited BGC-823 cell migration and invasion. (b) PX-12 treatment inhibited AGS cell migration and invasion. Representative images from triplicate experiments are shown. Magnification, ×200. The number of migrated and invaded cells is shown in the bottom panel. ^∗∗^ *P* < 0.01, ^∗∗∗^ *P* < 0.001.

The effects of PX-12 on human GC cell viability were examined using CCK-8 and plate colony formation assays. As shown in Figure 5(a), the BGC-823 and AGS cell viability was reduced in a dose- and time-dependent manner. Similarly, PX-12 treatment significantly reduced the colony formation of BGC-823 and AGS cells in a dose-dependent manner (Figure 5(b) **)**. To evaluate the effects of PX-12 on GC cell apoptosis, we used flow cytometry to detect cell apoptosis by annexin V-FITC/PI staining. The results showed that inhibition of Trx-1 by PX-12 induced BGC-823 and AGS cell apoptosis in a dose-dependent manner (Figure 5(c)). Cell migration and invasion were detected using a Transwell assay. PX-12 treatment inhibited the migration and invasion abilities of BGC-823 and AGS cells (Figures 6(a) and 6(b)).

### 3.6. Trx-1 Upregulates PTEN Expression and Activates AKT Signaling in Human GC Cells

**Figure 7:**
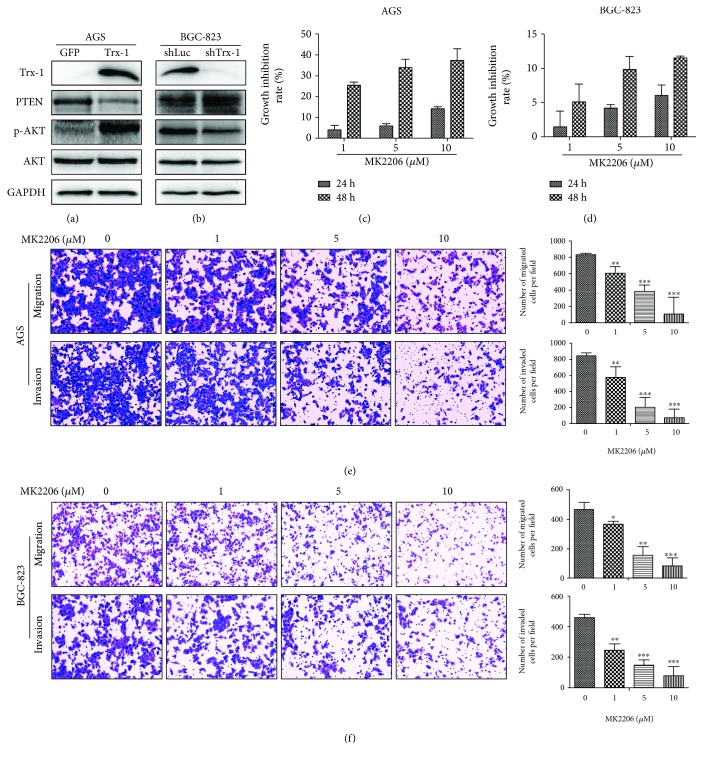
Trx-1 promotes gastric cancer cell growth, migration, and invasion through activation of AKT signaling. (a) Overexpression of Trx-1 increased phosphorylated AKT (p-AKT) and decreased PTEN expression levels in AGS cells. (b) Knockdown of Trx-1 expression decreased p-AKT and increased PTEN expression levels in BGC-823 cells. (c, d) Inhibition of AKT signaling pathway inhibited cell growth in AGS and BGC-823 cells. Cells were treated with AKT inhibitor MK2206 (1, 5, and 10 *μ*M) for 24 and 48 hours; CCK-8 assays were performed. (e, f) Inhibition of AKT signaling pathway inhibited AGS and BGC-823 cell migration and invasion. Representative images from triplicate experiments are shown. Magnification, ×200. ^∗^ *P* < 0.05, ^∗∗^ *P* < 0.01, ^∗∗∗^ *P* < 0.001.

To explore the potential molecular mechanisms of Trx-1's tumor promoting ability in GC, the expressions of PTEN and phospho-Akt (p-AKT) were detected by Western blotting when Trx-1 was overexpressed in AGS cells or silenced in BGC-823 cells. As shown in Figures 7(a) and 7(b), the overexpression of Trx-1 decreased PTEN and increased p-AKT expression levels, whereas the suppression of Trx-1 increased PTEN and decreased p-AKT expression levels. To further investigate the role of AKT signaling in GC, BGC-823 and AGS cells were treated with AKT inhibitor MK2206 (1, 5, and 10 *μ*M) for 24 or 48 hours. Inhibition of AKT signaling by MK2206 led to the growth inhibition of AGS (Figure 7(c)) and BGC-823 cells (Figure 7(d)). Furthermore, MK2206 treatment markedly decreased the migration and invasion of AGS and BGC-823 cells (Figures 7(e) and 7(f)).

## 4. Discussion

Although the increased expression of Trx-1 in various solid tumors has been reported, the prognostic significance of Trx-1 expression and function in human GC has not been extensively studied. Grogan et al. [17] studied the Trx-1 expression in paraffin-embedded tissue of 10 patients with primary high-risk gastric carcinoma using an immunohistochemical assay and found that Trx-1 was overexpressed in 8 out of 10 gastric carcinomas. Similarly, Noda et al. examined the Trx-1 expression in 42 human GC tissues and found that Trx-1 expression is elevated in tumor tissues and associated with poorly differentiated GC [16]. Lim et al. [28] detected Trx-1 and thioredoxin-interacting protein (TXNIP) mRNA expression levels in 68 stage III patients with GC using quantitative reverse transcription PCR and found the high Trx-1 and low TXNIP expression group exhibited a poorer prognosis than the other groups. Furthermore, they found that the Trx-1 protein was upregulated in 65% of the gastric cancer tissues by immunohistochemical staining. These findings are consistent with our results. However, these studies have a relatively small sample size. Our results showed that both increased mRNA expression and protein expression of Trx-1 were associated with poor patient survival in a relatively large sample size. We found that the mRNA expression of Trx-1 is increased in human GC tissues compared to the paired normal tissues in the GEO database. Patients with high Trx-1 mRNA expression levels had poor postoperative survival rates compared to patients with low Trx-1 mRNA expression levels (*P* = 0.0176). We also detected Trx-1 protein expression in 144 human GC tissues by immunohistochemistry. Kaplan-Meier analysis showed that patients with low expression levels of Trx-1 had longer overall survival than those patients with high expression levels (*P* < 0.001). Increased Trx-1 expression was significantly correlated with aggressive clinicopathological characteristics, including clinical stage, tumor stage, tumor size, and lymphatic metastasis. Moreover, multivariate analysis revealed that Trx-1 expression was an independent prognostic factor for overall survival of GC patients. Functional experiments indicated that silencing Trx-1 expression inhibited GC cell growth, migration, and invasion *in vitro* and tumor growth and lung metastasis *in vivo*. Conversely, overexpression of Trx-1 promoted GC cell growth, migration, and invasion.

The antitumor activity of the Trx-1 inhibitor, PX-12, has generated considerable interest in its use in a variety of solid tumors [29–32]. We previously reported that PX-12 treatment inhibits the growth of human colorectal cancer [33] and acute myeloid leukemia cells [34] via the induction of apoptosis. In addition, PX-12 inhibits colorectal cancer cell migration and invasion [33] and enhances the sensitivity of acute myeloid leukemia cells to arsenic trioxide [34]. The antitumor effect of PX-12 is also associated with cell cycle arrest, intracellular increases in ROS levels, and GSH depletion [35, 36]. PX-12 has already been assessed in phase II trials for the treatment of advanced pancreatic cancer and in a phase Ib trial for the treatment of advanced gastrointestinal cancers [15, 37]. In this study, we used PX-12 to inhibit Trx-1 in BGC-823 and AGS cell lines and found that PX-12 treatment significantly inhibited gastric cancer cell growth, migration, and invasion.

Trx-1 plays an essential role in maintaining a reduced environment in the cells via thiol-disulfide exchange reactions and protects against oxidative stress [38, 39]. The loss of redox homeostasis is involved in the pathogenesis and development of many diseases including GC [40]. The antioxidant functions of Trx-1 are also shown by involvement in DNA and protein repair by reducing ribonucleotide reductase and methionine sulfoxide reductases and modulating the activity of many redox-sensitive transcription factors [41]. In our study, we found that the overexpression of Trx-1 inhibited PTEN and increased p-AKT expression levels while the knockdown of Trx-1 resulted in increased PTEN protein expression and a reduction in AKT activity. The inhibition of AKT signaling pathway by MK2206 also inhibited GC cell growth, migration, and invasion. Being one of the most common tumor suppressors, PTEN influences cell survival and proliferation by regulating phosphatidylinositol 3-kinase protein kinase B (PKB/AKT) signaling [42]. Downregulation of PTEN promotes the migration and invasion in gastric cancer cells and overexpression of p-AKT is correlated with tumor progression and poor prognosis [43–46]. Based on these results, we speculate that Trx-1 may promote GC progression by activating AKT through PTEN.

## 5. Conclusions

In summary, Trx-1 was shown to be a prognostic marker for worse overall survival and to function as an independent prognostic factor in GC. Silencing of Trx-1 by lenti-shRNA could significantly inhibit GC cell growth and metastasis. Conversely, ectopic expression of Trx-1 promoted GC cell growth, migration, and invasion. Moreover, PX-12, a Trx-1 inhibitor, could also inhibit GC cell growth, migration, and invasion. Molecular mechanistically, Trx-1 could inhibit PTEN and increase p-AKT expression levels while downregulation of Trx-1 resulted in increased PTEN expression and a reduction in AKT activity in GC cells. Together, these results indicate that Trx-1 may be a promising prognostic indicator and therapeutic target for GC patients.

## Figures and Tables

**Table 1:** Trx-1 expression and clinicopathological parameters in gastric cancer specimens.

	All cases	Trx-1 protein		
	(*n* = 144)	High expression	Low expression	*P* value
*Sex*				*P* = 0.107
Male	45	16	29	
Female	99	50	49	
*Age (years)*				*P* = 0.871
<65	95	44	51	
≥65	49	22	27	
*Clinical stage*				*P* = 0.029^∗^
I–II	79	43	36	
III–IV	65	23	42	
*Differentiation grade*				*P* = 0.080^‡^
Well	1	0	1	
Moderate	58	22	36	
Poor	81	42	39	
Missing	4	2	2	
*Tumor stage*				*P* = 0.003^∗,‡^
N0/Ia	59	34	25	
N1/Ib	26	14	12	
N2/IIa	24	8	16	
N3/IIb	35	10	25	
*Tumor size (cm)*				*P* < 0.001^∗^
<3.4	66	41	25	
≥3.4	78	25	53	

^∗^χ^2^ test or Fisher's exact test. ^‡^Mann-Whitney *U* test (nonparametric). All missing values were excluded during statistical analyses. ^∗^ *P* < 0.05, which was considered statistically significant.

**Table 2:** Cox regression analysis of Trx-1 protein expression and clinicopathological covariates.

Characteristics	Univariate		Multivariate	
	HR (95% CI)	*P* value	HR (95% CI)	*P* value
Trx-1-high vs.Trx-1-low	5.56 (2.88–10.75)	**<0.001** ^∗^	5.44 (2.73–10.75)	**<0.001** ^∗^
Age (≥65 vs. <65)	1.44 (0.85–2.41)	0.174	1.26 (0.73–2.17)	0.414
Sex (male vs. female)	1.64 (0.97–2.76)	0.066	1.03 (0.60–1.76)	0.932
Clinical stage (III vs. I–II)	3.30 (1.92–5.67)	**<0.001** ^∗^	1.77 (0.81–3.86)	0.152
Higher differentiation grade	1.91 (1.10–3.29)	**0.021** ^∗^	2.58 (1.46–4.56)	**0.001** ^∗^
Advanced tumor stage	3.59 (1.90–6.78)	**<0.001** ^∗^	1.61 (0.67–3.91)	0.290
Tumor size (≥3.4 vs. <3.4)	3.21 (1.78–5.78)	**<0.001** ^∗^	1.70 (0.89–3.25)	0.108

HR: hazard ratio; CI: confidence interval. ^∗^ *P* < 0.05, which was considered statistically significant.

## Data Availability

The original research data used to support the findings of this study are included within the article and the supplementary information file. The gene expression data sets used to support the findings of this study are available on the Gene Expression Omnibus (GEO) database (https://www.ncbi.nlm.nih.gov/geo/).

## Abbreviations

AP-1: Action protein-1
ASK-1: Apoptosis signal-regulating kinase-1
CCK-8: Cell counting kit-8
FBS: Fetal bovine serum
GC: Gastric cancer
GEO: Gene expression ominous
GFP: Green fluorescent protein
IHC: Immunohistochemistry
p-AKT: Phosphorylated AKT
PCR: Polymerase chain reaction
PTEN: Phosphatase and tensin homologue
shLuc: shRNA targeting firefly luciferase
shTrx-1: shRNA targeting Trx-1
Trx-1: Thioredoxin-1.

## Supplementary Materials

Supplementary Materials Figure S1: knockdown of Trx-1 inhibits KATO III and AGS cell colony formation, migration, and invasion. (A) Trx-1 mRNA expression levels were detected by quantitative RT-PCR. (B) Trx-1 protein levels were detected by Western blotting. (C) The KATO III and AGS cell colony formation abilities were determined using the plate colony formation assay. (D) Cell migration and invasion were detected using a Transwell assay. Representative images from triplicate experiments are shown. Magnification, ×200. The quantitation of migrated and invaded cells is shown in the bottom panel. ^∗∗^ *P* < 0.01, ^∗∗∗^ *P* < 0.001.

## References

[1] Van Cutsem E., Sagaert X., Topal B., Haustermans K., Prenen H. (2016). Gastric cancer. *The Lancet*.

[2] Wadhwa R., Song S., Lee J. S., Yao Y., Wei Q., Ajani J. A. (2013). Gastric cancer-molecular and clinical dimensions. *Nature Reviews. Clinical Oncology*.

[3] Lin Y., Ueda J., Kikuchi S. (2011). Comparative epidemiology of gastric cancer between Japan and China. *World Journal of Gastroenterology*.

[4] Ferlay J., Soerjomataram I., Dikshit R. (2015). Cancer incidence and mortality worldwide: sources, methods and major patterns in GLOBOCAN 2012. *International Journal of Cancer*.

[5] Hoflehner E., Binder M., Hemmer W. (2012). Thioredoxin from the Indianmeal moth Plodia interpunctella: cloning and test of the allergenic potential in mice. *PloS One*.

[6] Csiki I., Yanagisawa K., Haruki N. (2006). Thioredoxin-1 modulates transcription of cyclooxygenase-2 via hypoxia-inducible factor-1*α* in non-small cell lung cancer. *Cancer Research*.

[7] Kim H. J., Chae H. Z., Kim Y. J. (2003). Preferential elevation of Prx I and Trx expression in lung cancer cells following hypoxia and in human lung cancer tissues. *Cell Biology and Toxicology*.

[8] Kim S. J., Miyoshi Y., Taguchi T. (2005). High thioredoxin expression is associated with resistance to docetaxel in primary breast cancer. *Clinical Cancer Research*.

[9] Zhao L., Li W., Zhou Y. (2015). The overexpression and nuclear translocation of Trx-1 during hypoxia confers on HepG2 cells resistance to DDP, and GL-V9 reverses the resistance by suppressing the Trx-1/Ref-1 axis. *Free Radical Biology & Medicine*.

[10] Mollbrink A., Jawad R., Vlamis-Gardikas A. (2014). Expression of thioredoxins and glutaredoxins in human hepatocellular carcinoma: correlation to cell proliferation, tumor size and metabolic syndrome. *International Journal of Immunopathology and Pharmacology*.

[11] Raffel J., Bhattacharyya A. K., Gallegos A. (2003). Increased expression of thioredoxin-1 in human colorectal cancer is associated with decreased patient survival. *The Journal of Laboratory and Clinical Medicine*.

[12] Lin F., Zhang P., Zuo Z. (2017). Thioredoxin-1 promotes colorectal cancer invasion and metastasis through crosstalk with S100P. *Cancer Letters*.

[13] Cha M. K., Suh K. H., Kim I. H. (2009). Overexpression of peroxiredoxin I and thioredoxin 1 in human breast carcinoma. *Journal of Experimental & Clinical Cancer Research*.

[14] Han H., Bearss D. J., Browne L. W., Calaluce R., Nagle R. B., Von Hoff D. D. (2002). Identification of differentially expressed genes in pancreatic cancer cells using cDNA microarray. *Cancer Research*.

[15] Ramanathan R. K., Abbruzzese J., Dragovich T. (2011). A randomized phase II study of PX-12, an inhibitor of thioredoxin in patients with advanced cancer of the pancreas following progression after a gemcitabine-containing combination. *Cancer Chemotherapy and Pharmacology*.

[16] Noda N., Ochiai A., Miyazaki K., Sugimura T., Terada M., Wakasugi H. (2000). Detection of thioredoxin in gastric cancer: association with histological type. *Antioxidants & Redox Signaling*.

[17] Grogan T. M., Fenoglio-Prieser C., Zeheb R. (2000). Thioredoxin, a putative oncogene product, is overexpressed in gastric carcinoma and associated with increased proliferation and increased cell survival. *Human Pathology*.

[18] Noike T., Miwa S., Soeda J., Kobayashi A., Miyagawa S. (2008). Increased expression of thioredoxin-1, vascular endothelial growth factor, and redox factor-1 is associated with poor prognosis in patients with liver metastasis from colorectal cancer. *Human Pathology*.

[19] Watanabe R., Nakamura H., Masutani H., Yodoi J. (2010). Anti-oxidative, anti-cancer and anti-inflammatory actions by thioredoxin 1 and thioredoxin-binding protein-2. *Pharmacology & Therapeutics*.

[20] Matthews J. R., Wakasugi N., Virelizier J. L., Yodoi J., Hay R. T. (1992). Thioredoxin regulates the DNA binding activity of NF-kappa B by reduction of a disulphide bond involving cysteine 62. *Nucleic Acids Research*.

[21] Hirota K., Matsui M., Iwata S., Nishiyama A., Mori K., Yodoi J. (1997). AP-1 transcriptional activity is regulated by a direct association between thioredoxin and Ref-1. *Proceedings of the National Academy of Sciences of the United States of America*.

[22] Hawkes H.-J. K., Karlenius T. C., Tonissen K. F. (2014). Regulation of the human thioredoxin gene promoter and its key substrates: a study of functional and putative regulatory elements. *Biochimica et Biophysica Acta (BBA) - General Subjects*.

[23] Saitoh M., Nishitoh H., Fujii M. (1998). Mammalian thioredoxin is a direct inhibitor of apoptosis signal-regulating kinase (ASK) 1. *The EMBO Journal*.

[24] Watson J. A., Rumsby M. G., Wolowacz R. G. (1999). Phage display identifies thioredoxin and superoxide dismutase as novel protein kinase C-interacting proteins: thioredoxin inhibits protein kinase C-mediated phosphorylation of histone. *The Biochemical Journal*.

[25] Zuo Z. G., Zhang P. L., Lin F. Y. (2018). Interplay between Trx-1 and S100P promotes colorectal cancer cell epithelial-mesenchymal transition by up-regulating S100A4 through AKT activation. *Journal of Cellular and Molecular Medicine*.

[26] Su E. J., Ernst L., Abdallah N. (2011). Estrogen receptor-*β* and fetoplacental endothelial prostanoid biosynthesis: a link to clinically demonstrated fetal growth restriction. *The Journal of Clinical Endocrinology & Metabolism*.

[27] Zhang P., Zuo Z., Wu A. (2017). miR-600 inhibits cell proliferation, migration and invasion by targeting p53 in mutant p53-expressing human colorectal cancer cell lines. *Oncology Letters*.

[28] Lim J. Y., Yoon S. O., Hong S. W., Kim J. W., Choi S. H., Cho J. Y. (2012). Thioredoxin and thioredoxin-interacting protein as prognostic markers for gastric cancer recurrence. *World Journal of Gastroenterology*.

[29] Li G. Z., Liang H. F., Liao B. (2015). PX-12 inhibits the growth of hepatocelluar carcinoma by inducing S-phase arrest, ROS-dependent apoptosis and enhances 5-FU cytotoxicity. *American Journal of Translational Research*.

[30] Baker A. F., Dragovich T., Tate W. R. (2006). The antitumor thioredoxin-1 inhibitor PX-12 (1-methylpropyl 2-imidazolyl disulfide) decreases thioredoxin-1 and VEGF levels in cancer patient plasma. *Journal of Laboratory and Clinical Medicine*.

[31] Welsh S. J., Williams R. R., Birmingham A., Newman D. J., Kirkpatrick D. L., Powis G. (2003). The thioredoxin redox inhibitors 1-methylpropyl 2-imidazolyl disulfide and pleurotin inhibit hypoxia-induced factor 1*α* and vascular endothelial growth factor formation. *Molecular Cancer Therapeutics*.

[32] You B. R., Shin H. R., Han B. R., Park W. H. (2015). PX-12 induces apoptosis in Calu-6 cells in an oxidative stress-dependent manner. *Tumour Biology*.

[33] Wang F., Lin F., Zhang P. (2015). Thioredoxin-1 inhibitor, 1-methylpropyl 2-imidazolyl disulfide, inhibits the growth, migration and invasion of colorectal cancer cell lines. *Oncology Reports*.

[34] Tan Y. X., Bi L. X., Zhang P. L. (2014). Thioredoxin-1 inhibitor PX-12 induces human acute myeloid leukemia cell apoptosis and enhances the sensitivity of cells to arsenic trioxide. *International Journal of Clinical and Experimental Pathology*.

[35] Shin H. R., You B. R., Park W. H. (2013). PX-12-induced HeLa cell death is associated with oxidative stress and GSH depletion. *Oncology Letters*.

[36] You B. R., Shin H. R., Park W. H. (2014). PX-12 inhibits the growth of A549 lung cancer cells via G2/M phase arrest and ROS-dependent apoptosis. *International Journal of Oncology*.

[37] Baker A. F., Adab K. N., Raghunand N. (2013). A phase IB trial of 24-hour intravenous PX-12, a thioredoxin-1 inhibitor, in patients with advanced gastrointestinal cancers. *Investigational New Drugs*.

[38] Flores L. C., Ortiz M., Dube S. (2012). Thioredoxin, oxidative stress, cancer and aging. *Longevity & Healthspan*.

[39] Takagi Y., Mitsui A., Nishiyama A. (1999). Overexpression of thioredoxin in transgenic mice attenuates focal ischemic brain damage. *Proceedings of the National Academy of Sciences of the United States of America*.

[40] Perez S., Talens-Visconti R., Rius-Perez S., Finamor I., Sastre J. (2017). Redox signaling in the gastrointestinal tract. *Free Radical Biology & Medicine*.

[41] Lu J., Holmgren A. (2014). The thioredoxin antioxidant system. *Free Radical Biology & Medicine*.

[42] Guo S. L., Ye H., Teng Y. (2013). Akt-p53-miR-365-cyclin D1/cdc25A axis contributes to gastric tumorigenesis induced by PTEN deficiency. *Nature Communications*.

[43] Ma J., Guo X., Zhang J. (2017). PTEN gene induces cell invasion and migration via regulating AKT/GSK-3*β*/*β*-catenin signaling pathway in human gastric cancer. *Digestive Diseases and Sciences*.

[44] Kobayashi I., Semba S., Matsuda Y., Kuroda Y., Yokozaki H. (2006). Significance of Akt phosphorylation on tumor growth and vascular endothelial growth factor expression in human gastric carcinoma. *Pathobiology*.

[45] Cinti C., Vindigni C., Zamparelli A. (2008). Activated Akt as an indicator of prognosis in gastric cancer. *Virchows Archiv*.

[46] Ang K. L., Shi D. L., Keong W. W., Epstein R. J. (2005). Upregulated Akt signaling adjacent to gastric cancers: implications for screening and chemoprevention. *Cancer Letters*.

