# Supplementary material for: Increased Thioredoxin-1 Expression Promotes Cancer Progression and Predicts Poor Prognosis in Patients with Gastric Cancer
Source: Oxid Med Cell Longev. 2019 Feb 18;2019:9291683. doi: 10.1155/2019/9291683 (PMC6398115; doi:10.1155/2019/9291683)
Supplement: Supplementary Materials — Figure S1: knockdown of Trx-1 inhibits KATO III and AGS cell colony formation, migration, and invasion. (A) Trx-1 mRNA expression levels were detected by quantitative RT-PCR. (B) Trx-1 protein levels were detected by Western blotting. (C) The KATO III and AGS cell colony formation abilities were determined using the plate colony formation assay. (D) Cell migration and invasion were detected using a Transwell assay. Representative images from triplicate experiments are shown. Magnification, ×200. The quantitation of migrated and invaded cells is shown in the bottom panel. ∗∗ P < 0.01, ∗∗∗ P < 0.001. [file 9291683.f1.docx]

**Supplemental figures**

**
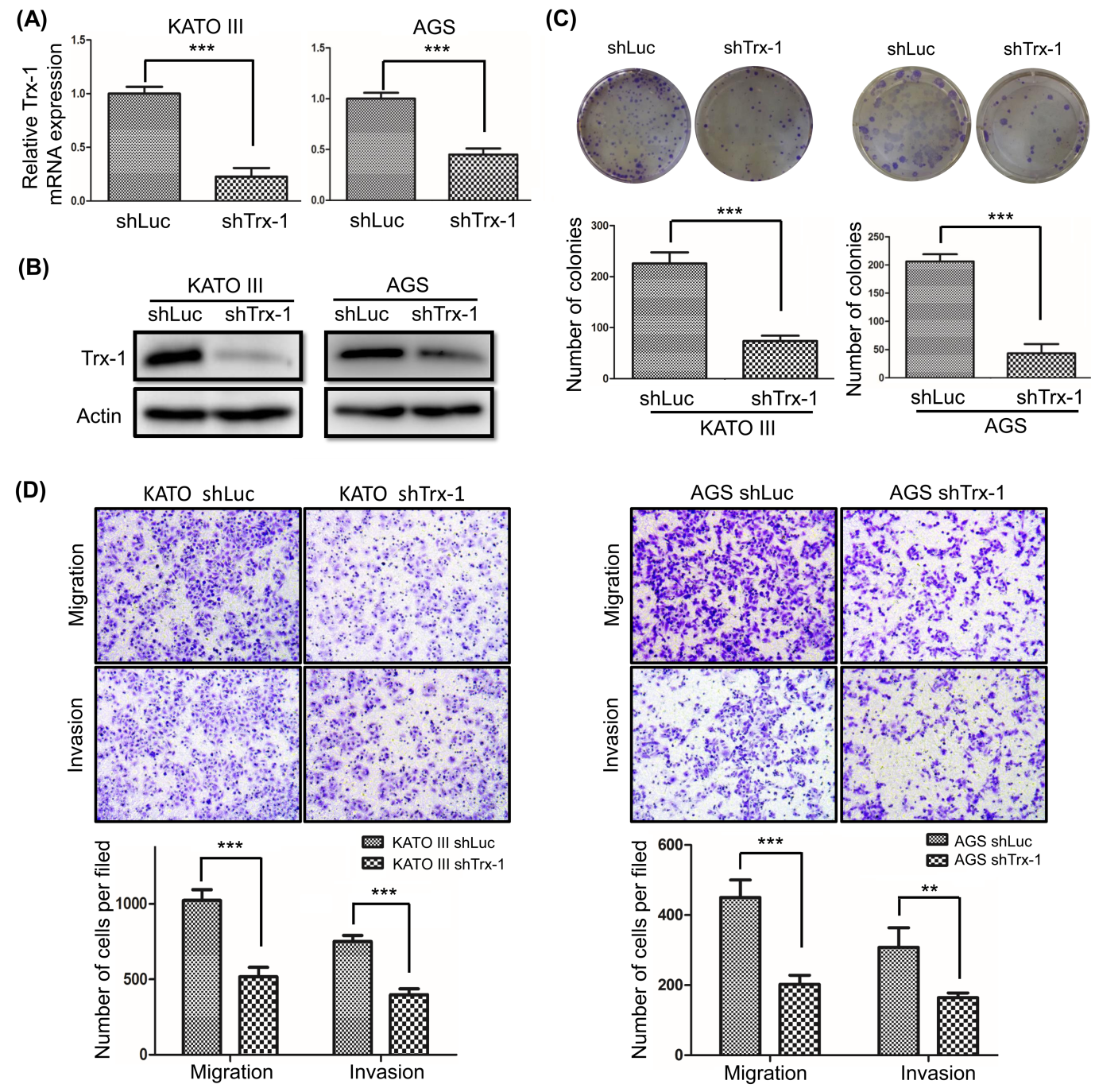
**

**Figure S1.** Knockdown of Trx-1 inhibits KATO III and AGS cell colony formation, migration and invasion. (A) Trx-1 mRNA expression levels were detected by quantitative RT-PCR. (B) Trx-1 protein levels were detected by Western blotting. (C) The KATO III and AGS cell colony formation abilities were determined using the plate colony formation assay. (D) Cell migration and invasion were detected using a Transwell assay. Representative images from triplicate experiments are shown. Magnification, ×200. The quantitation of migrated and invaded cells is shown on the bottom panel. ** *P* < 0.01, *** *P* < 0.001.
